# Association between hemoglobin, cerebral oxygenation and neurologic outcome in postcardiac arrest patients

**DOI:** 10.1186/cc14510

**Published:** 2015-03-16

**Authors:** I Meex, K Ameloot, C Genbrugge, M Dupont, B Ferdinande, J Dens, C Dedeyne

**Affiliations:** 1 ZOL Genk, Belgium

## Introduction

The safety of a restrictive transfusion threshold of 7 g/ dl applied in all critically ill patients can be questioned in postcardiac arrest (post-CA) patients since these are phenotypically clearly distinct. The aims of this study were to investigate the association between hemoglobin, cerebral oxygenation (SctO_2_) and outcome in post-CA patients.

## Methods

A prospective observational study in 82 post-CA patients during hypothermia in the first 24 hours of ICU stay. Hemoglobin was determined hourly together with a corresponding SctO_2_ by NIRS and SVO_2_ in patients with a pulmonary artery catheter (*n *= 62).

## Results

Based on 2,099 paired data, we found a strong linear relationship between hemoglobin and average SctO_2_ (SctO_2 _= 0.70 × hemoglobin + 56 (*R*^2 ^= 20.84, *P *= 10^-6^)). Given the previously suggested SctO_2_ target between 66 and 68%, hemoglobin levels below 10 g/dl generally resulted in suboptimal brain oxygenation. Forty-three patients (52%) had a good neurological outcome (CPC 1 to 3) at 180 days post CA. There was a significant association between average hemoglobin above 12.3 g/dl and good neurological outcome (OR = 2.88, 95% CI = 1.02; 8.16, *P *= 0.04). In a multivariate model, this association persisted after correction for comorbidities and age by the modified Charlson score (OR = 2.99, 95% CI = 1.05; 8.53, *P *= 0.03). This association was entirely driven by results obtained in patients with an average SVO_2 _below 70% (OR = 17.55, 95% CI = 1.67; 184.41, *P *= 0.01).

## Conclusion

There is a steep linear relationship between hemoglobin and SctO_2_ in post-CA patients with hemoglobin levels below 10 g/ dl generally resulting in cerebral desaturation. Average hemoglobin below 12.3 g/dl was independently associated with worse neurological outcome 180 days post CA. An interventional trial is necessary to investigate whether maintaining higher hemoglobin would improve outcome.

